# Author's Reply

**DOI:** 10.18502/ijrm.v19i6.9398

**Published:** 2021-07-27

**Authors:** Risikat Eniola Kadir, Abdulmumin Ibrahim, Balkis Abimbola Ibrahim, Sadiya Musa Gwadabe, Rukayat Sulaiman-Jaji, Munirat Foyeke Adigun, Adeoye Oyetunji Oyewopo

**Affiliations:** Department of Anatomy, Faculty of Basic Medical Sciences, University of Ilorin, Ilorin, PMB 1515, Nigeria; Department of Human Biology, University of Cape Town, Cape Town, South Africa

Thank you for the observations made, clarifications required as well as the suggestions. We have gone through and our response is thus:

1) We understand that spermatogenesis cycle in rats lasts on an average for 56 days. We administered rats with prednisolone, then treated them with various doses of *Vernonia amygdalina*, and a comparison was drawn between the groups and the control. This comparison demonstrates the differences. We treated the rats for 21 days, which has shown to make a difference in their spermatogenesis parameters when compared with the untreated group.. Similar studies with shorter duration of administration have also shown difference in these sperm parameters, even with as low as 14 days of treatment when compared to controls (1–3). In our study, the rats were immunosuppressed with prednisolone, and then treated with low and high doses of *Vernonia amygdalina* extract, to observe for the different possible effects. These groupings were ideas conceived by the authors, which was part of the experimental design of the study. Furthermore, studies have shown various administrations of similar and higher doses being used (4, 5). According to the published data by Akah et al. and Nwanjo, acute toxicity test in rats gave an LD${}_{50}$ of 1122 mg/kg as well as 1265.22 ± 56 mg/kg (6, 7).

2) We are aware of the relevance of assaying hormonal parameters when studying sperm and fertility analysis. We intend to go further with this in our future analysis.

3) There are various ways of obtaining plant extracts for experimental purpose. Your suggestion of using hydroalcoholic extract has been noted. We used aqueous extract as we know that this is the commonest manner in which the leaf is actually ingested. We therefore tried to simulate this usual manner in which the plant gets into the body. The plant leaves were washed, and then air dried prior to pulmerization. This we believe should to a large extent remove impurities. However, further studies will include fractionating the various compounds and testing these compounds individually.

4) The background to our study was to first note if there will be observable changes/effects following the administration of *Vernonia amygdalina* in immunosuppressed rats, a commonly consumed vegetable in our environment, which has been documented to have some beneficial properties. This was the first phase of the study. Subsequently, we will look at possible pathways through which these observed effects could occur. The hormonal and immunologic pathways will therefore be explored, as well as the possible roles of reactive oxygen species in this regard.

**Risikat Eniola Kadir^1^ M.Sc., Abdulmumin Ibrahim^1comma 2^ M.Sc., Balkis Abimbola Ibrahim^1^ B.Sc., Sadiya Musa Gwadabe^1^ M.Sc., Rukayat Sulaiman-Jaji^1^ M.Sc., Munirat Foyeke Adigun^1^ M.Sc., Adeoye Oyetunji Oyewopo^1^ Ph.D.**

^1^Department of Anatomy, Faculty of Basic Medical Sciences, University of Ilorin, Ilorin, PMB 1515, Nigeria.

^2^Department of Human Biology, University of Cape Town, Cape Town, South Africa.
